# Evaluation of a novel Serious Game based assessment tool for patients with Alzheimer’s disease

**DOI:** 10.1371/journal.pone.0175999

**Published:** 2017-05-04

**Authors:** Vanessa Vallejo, Patric Wyss, Luca Rampa, Andrei V. Mitache, René M. Müri, Urs P. Mosimann, Tobias Nef

**Affiliations:** 1 Gerontechnology and Rehabilitation Group, University of Bern, Bern, Switzerland; 2 University Hospital of Old Age Psychiatry and Psychotherapy, University of Bern, Bern, Switzerland; 3 Perception and Eye Movement Laboratory, Department of Neurology and Clinical Research, University Hospital Inselspital, University of Bern, Bern, Switzerland; 4 Private Hospital Wyss, Münchenbuchsee, Switzerland; 5 ARTORG Center for Biomedical Engineering Research, University of Bern, Bern Switzerland; Taipei Veterans General Hospital, TAIWAN

## Abstract

Despite growing interest in developing ecological assessment of difficulties in patients with Alzheimer’s disease new methods assessing the cognitive difficulties related to functional activities are missing. To complete current evaluation, the use of Serious Games can be a promising approach as it offers the possibility to recreate a virtual environment with daily living activities and a precise and complete cognitive evaluation. The aim of the present study was to evaluate the usability and the screening potential of a new ecological tool for assessment of cognitive functions in patients with Alzheimer’s disease. Eighteen patients with Alzheimer’s disease and twenty healthy controls participated to the study. They were asked to complete six daily living virtual tasks assessing several cognitive functions: three navigation tasks, one shopping task, one cooking task and one table preparation task following a one-day scenario. Usability of the game was evaluated through a questionnaire and through the analysis of the computer interactions for the two groups. Furthermore, the performances in terms of time to achieve the task and percentage of completion on the several tasks were recorded. Results indicate that both groups subjectively found the game user friendly and they were objectively able to play the game without computer interactions difficulties. Comparison of the performances between the two groups indicated a significant difference in terms of percentage of achievement of the several tasks and in terms of time they needed to achieve the several tasks. This study suggests that this new Serious Game based assessment tool is a user-friendly and ecological method to evaluate the cognitive abilities related to the difficulties patients can encounter in daily living activities and can be used as a screening tool as it allowed to distinguish Alzheimer’s patient’s performance from healthy controls.

## Introduction

Alzheimer’s disease (AD) is the most common form of dementia and is characterized by a decline in cognition, impaired social behaviour and decline in instrumental activities of daily living (iADL) [1]. Instrumental daily living impairments due to cognitive decline appear to emerge early in the course of Alzheimer’s disease [2] and even constitute an early marker of the disease [3]. Additionally, the disease becomes often apparent at a functional level, that is the ability to cope with these instrumental activities of daily living, like for example using the telephone or taking public transport [4]. These activities are crucial to keep an independent life and as the disease progresses towards more important severity stages, the risk of institutionalisation increases [5]. To address this issue, ecological assessment of iADL is crucial for better intervention process, in order to precisely know the kind of difficulties in daily living tasks. While the questionnaire-based evaluation with performance-based scale is a well- established measure for assessment of activities of daily living through the subjective view of the patient or the caregivers [6–9], a real evaluation of ecological tasks offer the advantage of being more realistic and allow an objective measure of these activities. Furthermore, by adding real tasks in evaluation and putting patients in the situation where they can meet difficulties in everyday life, it offers them the possibility to perceive the motivation of this evaluation and anticipates their needs at home. However, the implementation of real evaluation of activities of daily living reminds unusual and difficult to achieve due to the needs of special and expensive infrastructures [10].

Additionally, as the impairments in these activities are closely related to the cognitive decline, a comprehensive assessment of cognitive abilities is also essential. While a plethora of studies have documented the ecological evaluation of executive functions related decline across a wide range of cognitive functions and domains [11–14] usually at single process level, their combined consequences on everyday abilities has been scarcely investigated. Yet again, the implementation of such evaluation requires certain exigency with regards to the material employed. To assess this exigence, there is a growing evidence that virtual reality and computers games with serious purposes, so called Serious Games, represent a good approach to complete the current evaluations [15]. Serious Games have the advantages of offering the possibility to recreate a functional environment where patients can encounter difficulties similar to the one in real life [16]. Moreover, authors have shown that it is a valid evaluation instrument for iADL [17] and also for cognitive screening [18]. It also offers other advantages regarding the technical parameters, i.e. record of complete performance, ease of administration [19] and regarding the motivation and fun generated while playing the game [20,21]. Several authors have investigated the challenges in terms of usability when it comes to the development of a game targeted for patients with AD [22–24]. Since the population of old adults or patients with AD have generally little or no computer experience, performances in the game can be influenced by using these technologies, thereby not representative enough of real performances. Thus, the criteria for developing tools for older adults and patients with cognitive deficits have to be fulfilled in order to evaluate the performances themselves and not be influenced by computer-interactions difficulties. According to Bouchard et al. [23], in-game challenges, appropriate interaction mechanisms, adequate assistive prompting and effective visual and auditory assets are the keys guidelines for designing effective serious games for old adults and cognitively impaired people.

Following these guidelines, we developed a daily living scenario with six tasks to investigate the nature of difficulties associated with functional impairment in daily living activities. The aim of the present study was to evaluate the usability and the screening potential of a new Serious Game based assessment tool for patients with AD.

## Materials and methods

### Participants

Twenty healthy controls with no indication of cognitive impairment and eighteen patients with AD participated in this study. The healthy control group was recruited from the Seniors University of Bern, Switzerland. They were assessed with the Montreal Cognitive Assessment (MoCA) screening tool [25]. The corresponding inclusion criterion for the control group was a cut-off score above 26 in the MoCA and no psychiatric or neurological disorders. Patients eligible for the study were recruited from the Interdisciplinary Memory Clinic of the University Hospital of Old Age Psychiatry in Bern, Switzerland, and were all previously diagnosed with probable AD, according to the International Classification of Diseases, 10^th^ Revision. Patients were assessed with the German version of the CERAD neuropsychological battery (Consortium to Establish a Registry for Alzheimer’s Disease; [26]). Additional clinical scales were also administered for the evaluation of the activities of daily living: the Bristol Activities of Daily Living Scale [6], and the Functional Activities Questionnaire [27]. Finally, the Geriatric Depression Scale [28] was administrated to verify the presence and severity of depressive symptoms. Structural MRI data were also evaluated for all patients, in order to exclude any other brain pathology.

Exclusion criteria for both groups were psychiatric disorders and insufficient or insufficiently corrected visual acuity and inability to understand the instructions.

The experiment was carried out in accordance with the latest version of the Declaration of Helsinki. All participants and the responsible caregivers of the patients with AD gave written informed consent prior to participation to the study which was approved by the Ethical Committee of the State of Bern.

Demographics and clinical characteristics of the participants are summarized in Table 1.

**Table 1:** Demographics and clinical characteristics of the participants (mean ± SD).

	Control subjects (N = 20)	AD patients (N = 18)
Age (years)	74.6 ± 5.9	77.8 ± 6.2
Education (years)	12.1 ± 3.4	11.6 ± 2.6
Gender (male:female)	12:8	9:9
*Global Cognition*		
MoCA	29 ± .8	19.5 ± 2.8*

Abbreviation: MoCA, Monreal Cognitive Assessment.

*Differs from healthy controls (P < .001).

### Serious game scenario

**Fig 1:**
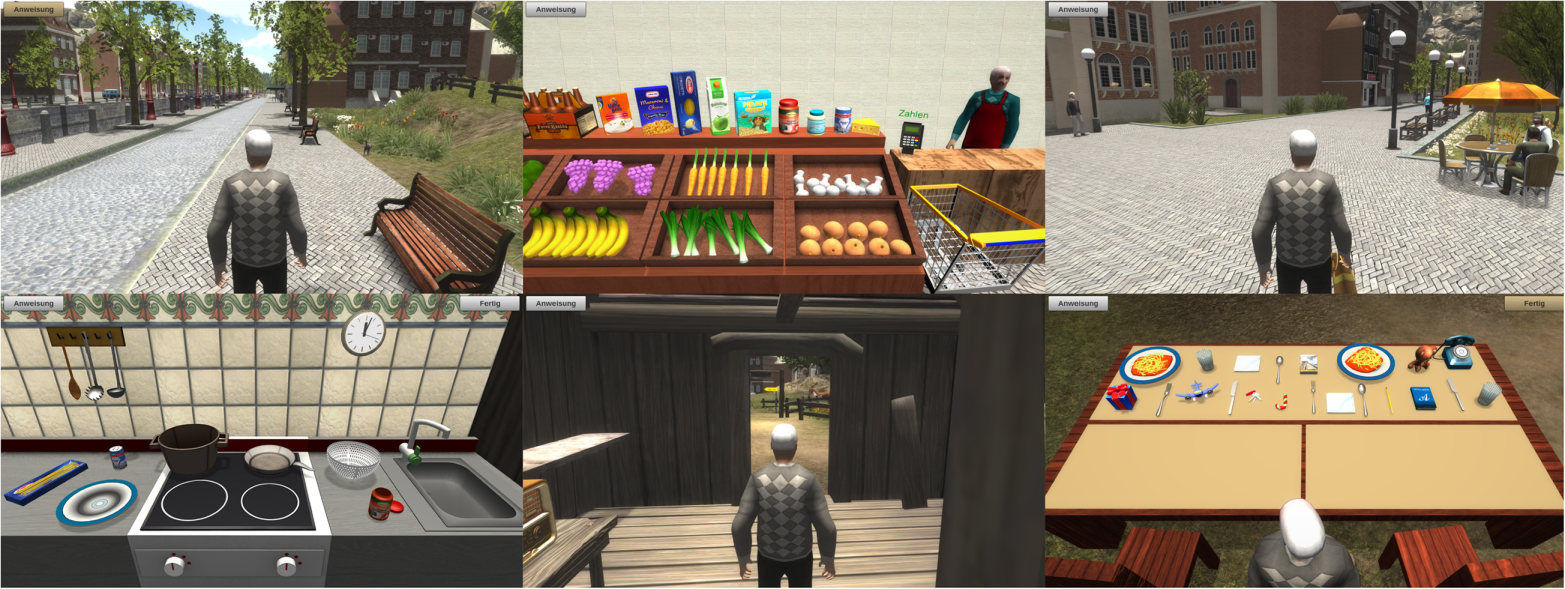
Screen shots of the six virtual tasks. (A) The navigation task “Go to the shop”, (B) The shopping task, (C) The navigation task “Go back home”, (D) The cooking task, (E) The navigation task “Go to the garden”, (F) The table preparation task.

The Serious Game consisted of a simulation of daily living situations with six tasks, i.e. three navigation tasks, a shopping task, a cooking task and a table preparation task (Fig 1). These tasks were used to examine episodic and prospective memory, visuo-spatial orientation, executive functions, attention and general processing speed. Table 2 provides a description of the tasks and actions with the outcomes measures and the main cognitive functions tested by these actions.

**Table 2:** Descriptions of tasks and respectively outcomes measures and main cognitive functions assessed.

Tasks and actions	Outcome measures	Cognitive functions assessed
**Task 1: Navigation “Go to the Shop”** *Participants have to navigate through the virtual environment*, *following the arrows to find the way to the shop*	Time on the task 1 Time spent to search the way (inactivity time)	Visuo-spatial orientation
**Task 2: Navigation “Go back home”** *Participants have to remember the way to go home*	Time on the task 2 Task completion: way correctly remembered	Spatial memory
**Task 3: Shopping** *Participants have to learn a list of three ingredients and recognize them once arrived in the shop*	Time on the task 3 Task completion: correct ingredients collected	Episodic memory
**Task 4: Cooking** *Participants have to prepare a plate with pasta and tomato sauce*	Time on the task 4 Task completion: food correctly prepared	Executive functions and attention
**Task 5: Navigation “Go to the garden”** *Participants have to remember to go to the garden to prepare the table*	Time on the task 5 Task completion: go to the garden without asking for the request button	Prospective memory
**Task 6: Table preparation** *Participants have to prepare a table for two persons*	Time on the task 6 Task completion: table correctly prepared	Executive functions and attention

Participants had to navigate in the virtual environment to find the market by following arrows on the way and remember the way for coming home. They also had a small navigation task to go to the garden from inside the house. The shopping task consisted on collecting three ingredients previously remembered; participants could move and use the several objects on the screen for the cooking task in order to prepare a plate of spaghetti with tomato sauce. Finally, the table preparation task consisted on moving and placing the objects presented on the screen to set a table for two persons. In case they didn’t remember what to do in the game, they always had the possibility to press a “Instructions” button permanently available at the top left of the screen.

While participants performed the tasks, the system recorded several parameters: the duration time for each task, the time spent to search the way to go to the shop (i.e. the inactivity time) and the correctness of the task achievement for the navigation tasks “Go back home” and “Go to the garden”, for the shopping task, the cooking task and the table preparation. The navigation task “Go back home” was correctly achieved if participants correctly remembered the way to go home and the navigation task “Go to the garden” was correctly achieved when participants went to the garden without asking for the instructions request. The shopping task was correctly achieved if participants remembered to buy the three ingredients (i.e. pasta, tomato sauce and salt) without including any other distractors ingredients, the cooking task was achieved when the pasta and tomato sauce were cooked and placed in the plate, and finally the table preparation was correctly realized when plates with cutlery without distractors were placed equally for two persons.

**Fig 2:**
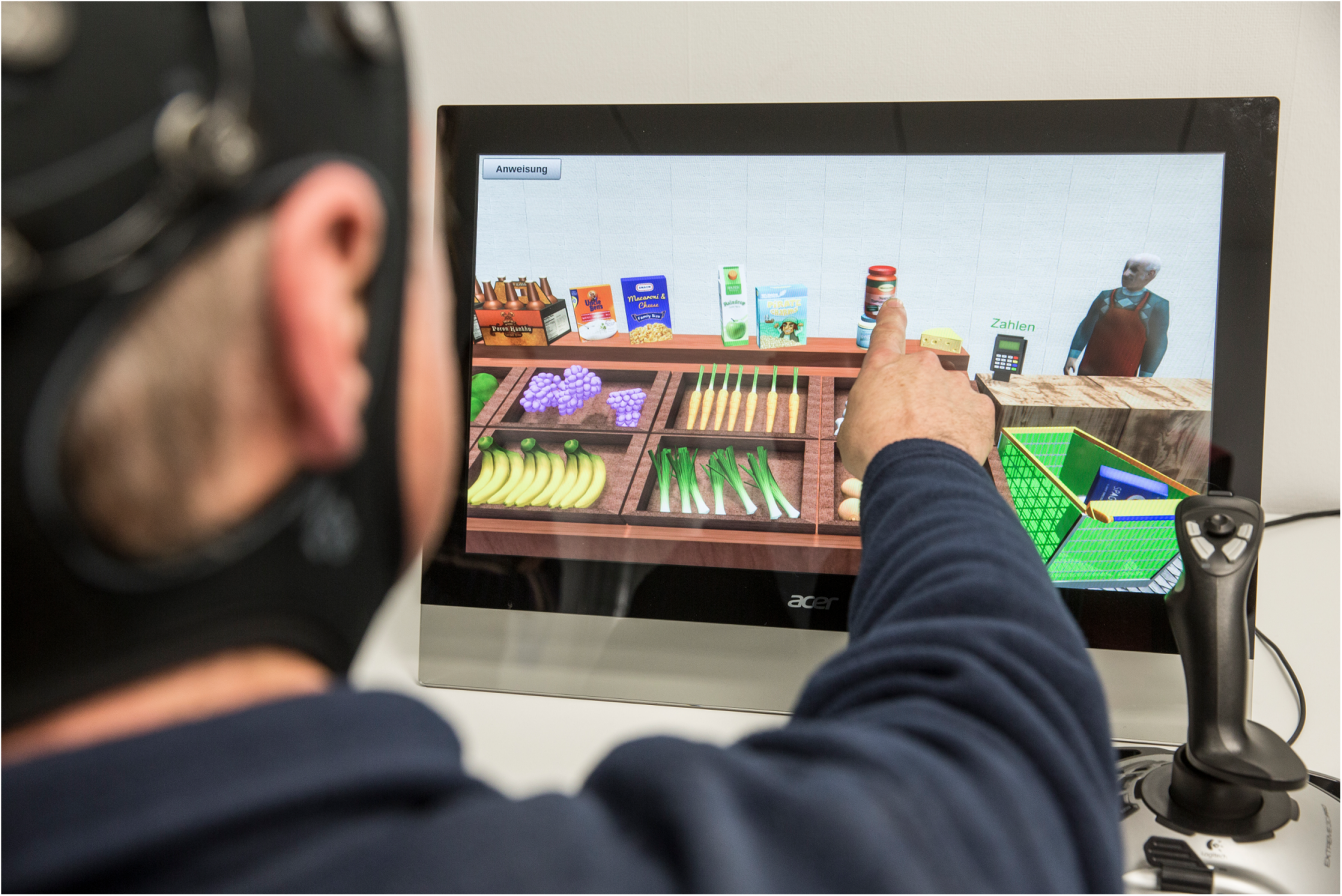
Participant during the experiment, picking an ingredient during the shopping task. (Participant on the picture gave written informed consent to be photographed for publication).

The virtual screening system was developed at the Gerontechnolgy and Rehabilitation Group, ARTORG Center for Biomedical Engineering Research, University of Bern, using Unity 3D. While playing, the in-game events were continuously collected and stored on disk in JavaScript Object Notation (JSON) format. The game was presented on a Touch Screen (21" Asus screen, 1920x1080, ASUSTeK Computer, REPUBLIC OF CHINA). The touch-interface was used for the shopping, cooking and table-preparation tasks. A Joystick (Logitech, Lausanne, VD, SUISSE) (Fig 2) was used for the navigation task.

The computer interactions for measure of objective usability were recorded in order to exclude all performances due to the usability aspect; the interaction with the joystick was examined by analyzing the deviation path compared to the ideal and most direct path to go to the shop and to come back. The velocity, the length and the curvature of the movements with the touch screen, as well as the number of movements needed to achieve the task were analyzed.

### Procedure

All participants first filled a questionnaire including demographic information, personal and medical history. They then completed a training in the game in order to get familiarize with the interactions of each tasks. The instructions were given to the participants before the game onset, and participants were asked to achieve the task as they would normally do in real life. They started the virtual tasks following a story line where they first had to learn the three ingredients appearing on the screen (episodic memory, learn), then go to the shop by following arrows on the way (visuo-spatial orientation), buy only the three ingredients in the shop (episodic memory, recognition), go back home using the same way (spatial memory), cook a plate of pasta with tomato sauce (executive functions and attention) and finally remember to go to the garden (prospective memory) and prepare a table for two persons (executive functions and attention). Participants were then asked to repeat the guidelines to check that they correctly encoded all the instructions before starting the game.

Finally, for a subjective measure of usability, participants responded to a questionnaire about the game, the System Usability Scale (Brooke, 1996), in order to evaluate the usability of the game, answering then affirmations.

### Statistical analysis

Statistical analyses were performed using R (R Foundation for Statistical Computing, Vienna, Austria) for the analysis with response variable task achievement and SPSSv20 (IBM Corporation, Armonk, NY, USA) for all the other analysis. To control for a possible relation between level of education and performance in the game, a logit model was computed. To analyse the Serious Game outcomes, independent-samples t-tests were used for group comparison for the time for each task and the time spent to search the way. To examine possible difference between the two groups in the binary response variable task achievement, separate logit-models per task were computed. To test for significant group differences, we used the likelihood-ratio-test.

An independent-samples t-test was also used to assess potential differences in the objective measures of usability (i.e. differences in velocity, curvature, length of movements, and number of movements). As the subjective measure dependent variable was measured in an ordinal level, including a Likert Scale, a non-parametric analysis of variance (Friedman’s ANOVA) was used to compare the mean score of usability with the System Usability Scale.

The significance level was set α < .05 for all analyses.

## Results

**Fig 3:**
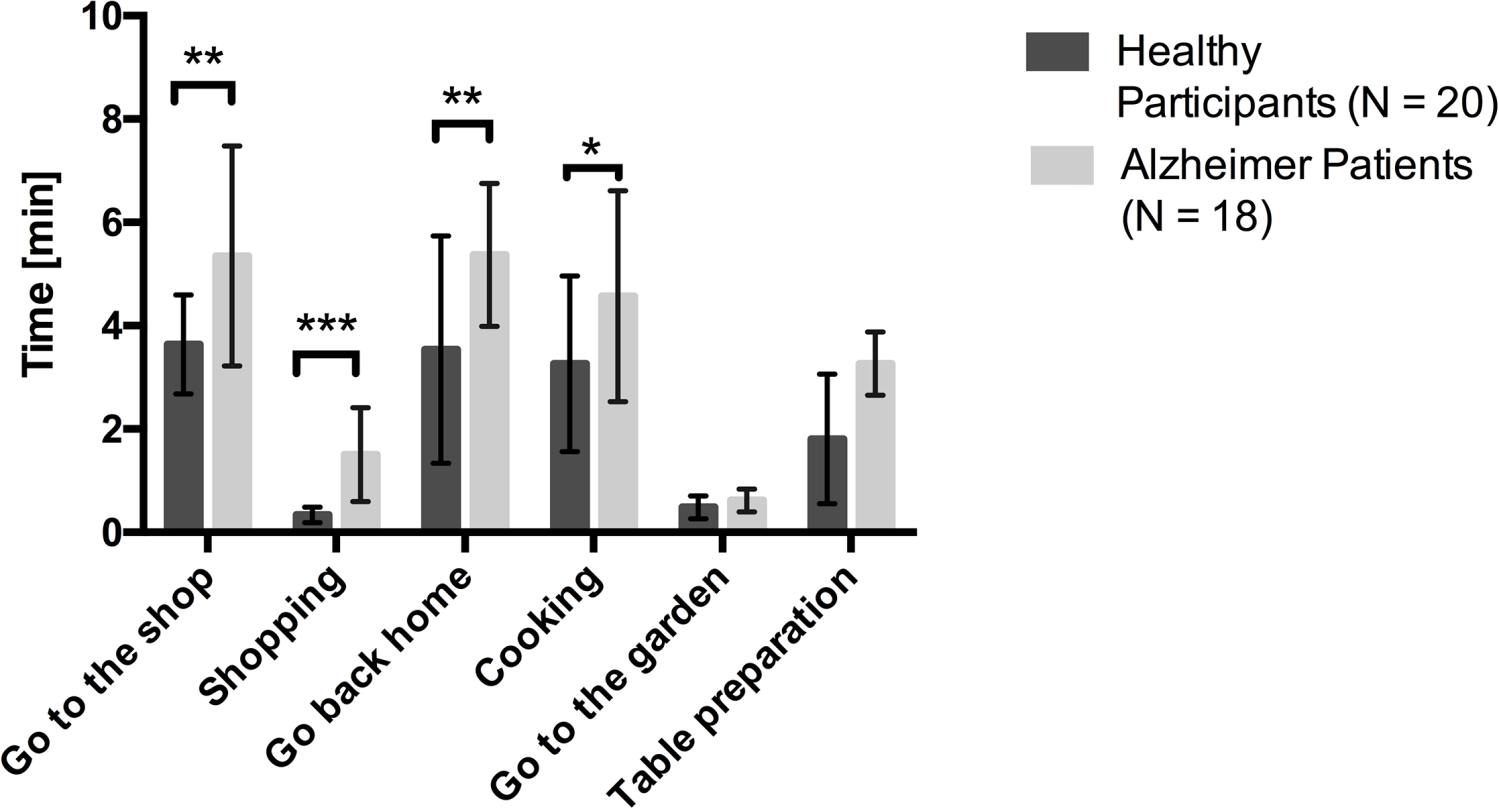
Mean time to achieve the several tasks for the two groups with the standard deviation. * p < .05, ** p < .01, *** p < .001.

The logit model did not reveal any relation between the level of education and performance of this assessment (*χ*^2^(1) = 2. 61, *p* > 0.05) suggesting that the level of education doesn’t have an influence on the performance in this assessment. Fig 3 shows group differences in the mean time for each task in the order of the storyline, in which they had to achieve the several tasks. The independent-samples t-tests yielded a significant group difference in the mean time for the navigation tasks “Go to the Shop” (t (36) = 12.457, p = .003), “Go back home” (t (36) = .054, p = .005), whereas no significant differences were found for the navigation task “Go to the garden” (t (36) = .001, p = .069) between the two groups. Furthermore, it revealed a significant group difference in the mean time for the shopping task (t (36) = 14.731, p < .001) and the cooking task (t (36) = 1.265, p < .05), indicating that patients needed more time to achieve these tasks compared to healthy controls, while no differences between the two groups were established for the mean time of the table preparation task (t (36) = 2.798, p = .321).

**Fig 4:**
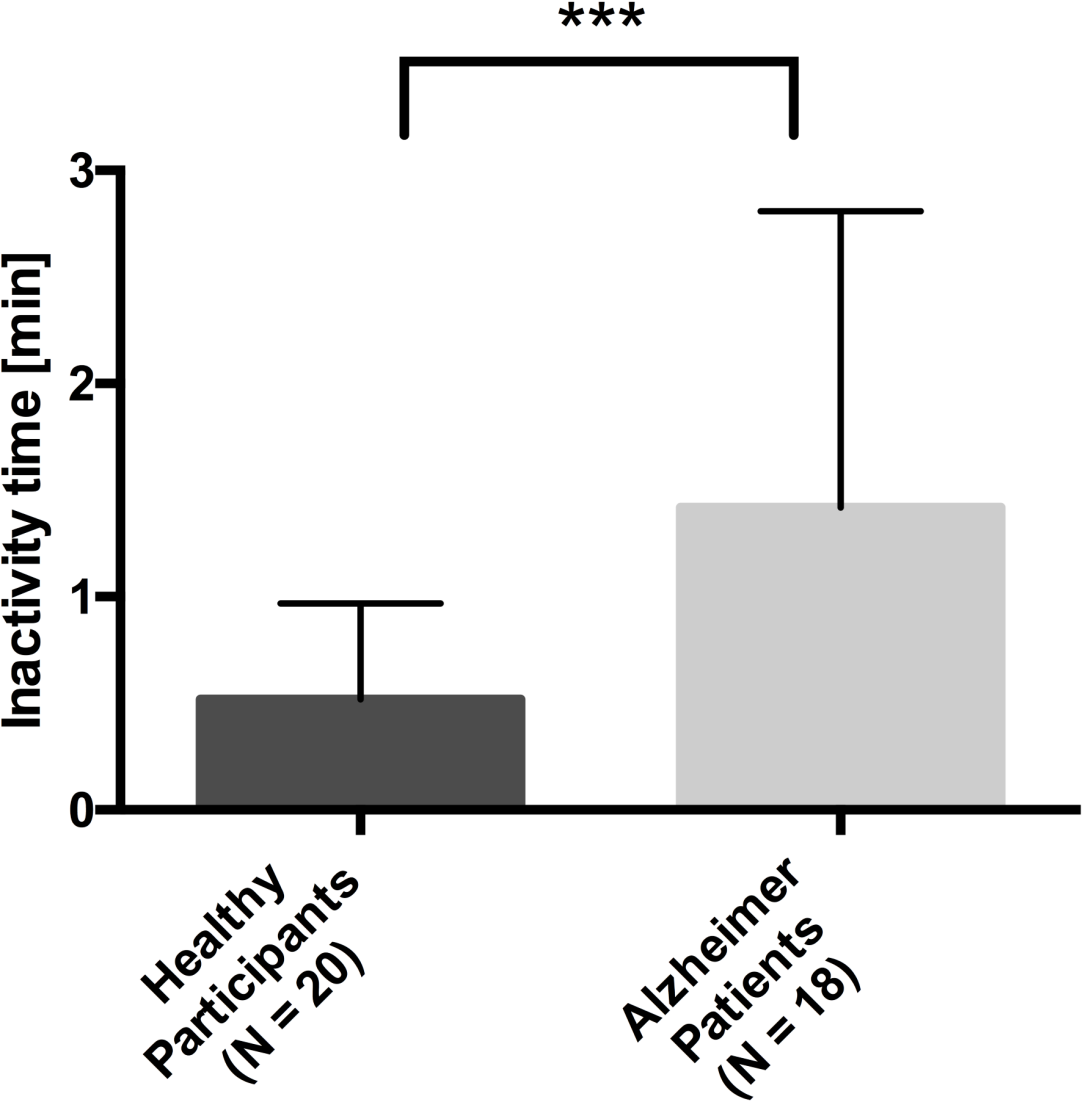
Mean time to search the way for the two groups with the standard deviation. * p < .05, ** p < .01, *** p < .001.

The independent-samples t-tests on the time to search the way yielded a significant difference between the two groups (t (36) = 6.760 p = .01), signifying that patients needed significantly more time to search their way compared to the healthy controls (Fig 4).

**Fig 5:**
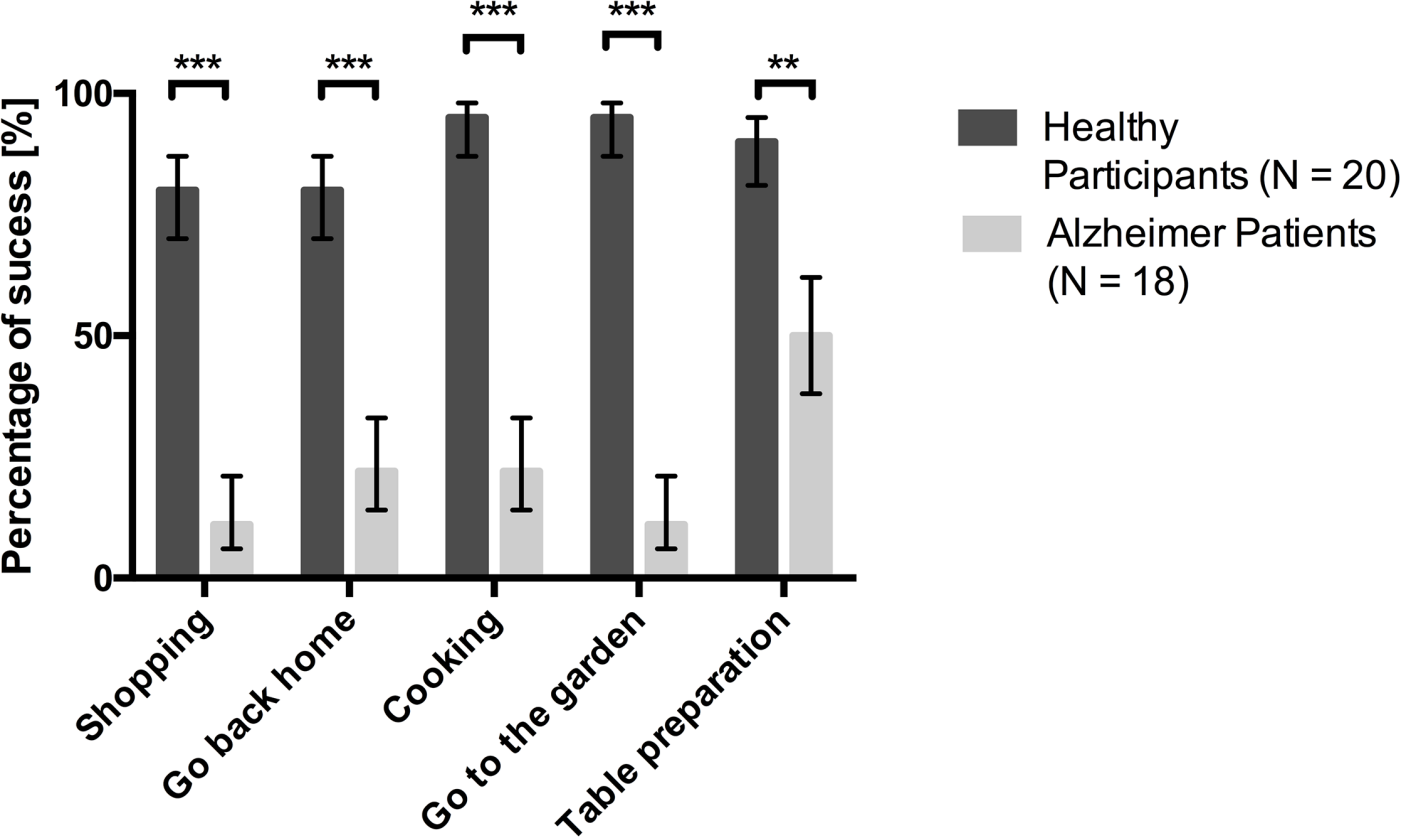
Fitted probabilities of achievement for both groups including the bars based on the standard errors (SEs) of the logits * p < .05, ** p < .01, *** p < .001.

Finally, the logit-models revealed a significant difference between the two groups in the probabilities of achieving the cooking task (*χ*^2^(1) = 23.97, *p* < .001), the shopping task (*χ*^2^(1) = 20.00, *p* < .001), the navigation tasks "“Go back home” (*χ*^2^(1) = 13.49, *p* < .001) and "Go to the garden" (*χ*^2^(1) = 31.76, *p* < 0.001) and the table preparation task (*χ*^2^ = 7.77, *p* < .01) (Fig 5).

Table 3 shows group differences for the objective and subjective measure of usability. Regarding the objective measure of usability, the independent-samples t-tests did not yield any significant differences between patients and healthy controls in the curvature (t (36) = .024, p = .479), in the velocity (t (36) = .035, p = .930), in the length of movements (t (36) = 2.176, p = .278), and number of movements (t (36) = 4.017, p = .807). Hence, the nature of interactions was equivalent in healthy controls and AD patients, and the difference of performance was not influenced by the usability. The Friedman’s ANOVA did not reveal any significant effect of score (p = .806), indicating that both group found the game user–friendly.

**Table 3:** Results for the objective and subjective measure of usability (mean ± SD).

	Control subjects (N = 20)	AD patients (N = 18)
*Objective measure of usability*		
Curvature (R coefficient)	.82 ± .08	.80 ± .074
Velocity (mm/Sec)	232.11 ± 83.57	234.60 ± 89.87
	507.03 ± 190.89	446.63 ± 139.92
Number of movements	27.80 ± 10.64	29.17 ± 22.16
*Subjective measure of usability*		
SUS Score (percentage)	83.5 ± 11.16	83.75 ± 9.82

Abbreviation: SUS, System Usability Scale

## Discussion

The main objective of the current study was to develop a new tool for assessment of cognitive functions in patients with AD by using an ecological approach involving several daily living activities and to evaluate the usability and the assessment potential of this tool. The comparison of the performance between the two groups indicated a significant difference in terms of percentage of achievement of the several tasks and in terms of time needed to achieve the subtasks. Results for the usability measures indicate that patients and healthy participants found the game user-friendly and they were objectively able to play without computer interaction difficulties.

Development of new technology exploring human cognition in real-world environments has expended [29–31]. However, few studies using virtual reality have analysed the direct effects of AD on real-world activities [32,33]. The present results showed a significant difference between the healthy group and the patients with AD in performances in the several activities of daily living. If this difference was expected, the qualitative additional information regarding the errors in achievement of the several activities brings crucial information in terms of cognitive functions needed to achieve the tasks and in terms of difficulties patients with AD can encounter in real life [34]. For example, in the navigation task “Go back home”, most of the patients couldn’t remember the way they took to go to the shop and got lost in the virtual environment. In daily living, these deficits have life-threatening complications that emerge from wandering and getting lost [35]. Navigational impairment is a key feature of AD [36] and appears from deficits in associating visual scenes and locations [37]. By adding such a navigational task in a rehabilitation tool for example, deficits in orientation in new location with unfamiliar situations can be trained in a safe, daily living environment. Regarding the behavior and the difficulties patients had in the shopping task, they compensated their deficits in remembering the three ingredients by adding everything they found in the shop, or on the contrary picked only one ingredient. For the cooking task, most of the patients showed a variety of errors, including breaking rules (e.g. put the pasta without water), leaving items unfinished (e.g. let the colander with pasta in the sink), and forgetting to carry out prospective memory items (e.g. forgot to turn off the cooktop). These errors are similar to the one found by Shallice and Burgess [38] in patients with brain injury on everyday multitasking tasks. In their study, participants were asked to achieve a number of errands in a shopping zone while following a set of rules. According to the errors patients committed, the authors concluded that the patients had difficulties implementing and keeping track of their intentions to switch to other tasks. Finally, in the table preparation, patients mostly forgot to set the table for one person or added distractors while preparing the table. This additional information can support patient-specific rehabilitation processes. It allows targeting the individual difficulties in daily living, while bringing crucial additional information to the current neuropsychological tests or questionnaires used for the evaluation of these patients. As pointed out by Pérès [3], the cognitive impairment measured using neuropsychological tests is the manifestation of the disease only in an experimental situation, whereas difficulties in iADLs are the expression in the real situation of daily life. Additionally, the responses to the questionnaires measuring iADLs bring the information of the presence or absence of difficulties [39], while the current serious game based assessment tool can bring further information regarding the nature of these difficulties.

Also time indications and time of completion differences among participants can be a good indicator of the performance in the task [40]. While patients needed more time to complete the shopping task, the cooking task and the two navigation tasks “go to the shop” and “come back home”, no differences were found for the table preparation task and for the navigation task “Go to the garden”. We can explain this absence of difference as these tasks were easier to achieve in terms of cognitive processes required. Indeed, the navigation task “Go to the shop” involved spatial navigation ability in a more complex environment as patients needed to search for a longer way to find the shop, and the navigation task “Go back home” was subtended by spatial memory. However, the navigation task “Go to the garden” involved a shorter way, i.e. just to navigate outside the house, towards the table, which embraces easier spatial abilities without involving any spatial memory to remember the way. The same is true for the table preparation which requires less planning skills and less multiple shifts between tasks that can occur when preparing a meal for example [41]. The assessment of cognitive abilities in a range of several tasks, from accomplishing an easy task involving single cognitive processes, to accomplish more complex tasks involving several cognitive processes acting together is of major importance when developing an intervention for patients with AD. As highlighted by Fissler et al. [42], the idea of combining novel interventions (i.e. using daily living activities) with process based cognitive training brings the advantage of having high task variability overlapping in targeted processing demands and based the rehabilitation on impact cognitive functions to help the impaired cognitive processes.

Creating a Serious Game for Alzheimer’s disease requires several criteria to be satisfied in order to have a functional, enjoyable and user–friendly game [43]. According to Steinberg [44], the gold standard for interacting with a virtual environment is an intuitive interface without any need of training or previous experience, as it motivates the participants without experience to interact with the device. Moreover, developing a gaming tool aimed at cognitive testing requires additional criteria then just playing for fun; it requires a precise evaluation of the cognitive abilities to complete the tasks. Therefore, it is essential to ensure that the achievements in the game are due to the performances themselves and not due to any computer interactions difficulties that might negatively influence the evaluation of these cognitive performances. In the present study, no differences were found between the two groups in the objective measure of usability, confirming that the differences of performances in the game are due to the performances in the tasks and not to any possible difficulties of the participants in computer interactions. Analyzing precise interactions with computers bring important information regarding the usability of a device, in particular when it comes to older adults and patients with AD, with a decline in physical aptitude in fine motor control [45]. The current findings are in line with previous results showing that the joystick and the touchscreen were well accepted by participants without computer experience [46,47]. Considering the results in the usability questionnaire, both group found the game user friendly, which indicates that also subjectively they enjoyed playing the game and didn’t meet any problems to interact with it. These findings support the use of virtual environment testing in patients even with no experience with computers for assessment or training of cognitive functions.

In conclusion, the current study provides a first valuable insight into the use of a new user–friendly tool evaluating cognitive functions associated to the nature of the functional deterioration in patients with AD. Futures studies should address this question in a larger sample size including the evaluation of cognitive battery linked to the performances of the participants in the serious game. Another limitation of the study is the nature of the daily living tasks used in this tool, which can be considered as culture-oriented and gender-oriented tasks. This culture- dependent scenario might limit the global usability of the software and culture specific variants will have to be further developed. Yet, the choice of these daily living tasks were based on the instrumental activities assessed in the scale developed by Lawton and Brody [48], considered as a cross-cultural scale and used to assess the functioning in these iADL in multi-ethnic populations [49–52]. Additionally, with relevance of clinical care, the evaluation of difficulties in such tasks is crucially important to keep an independent life, despite other more satisfactory gender neutral tasks. Research on early detection of dementia should provide new instruments for cognitive and functional evaluation that are sensitive enough to detect early manifestations of the disease [3]. This also involves strategies for analysis of the difficulties in order to develop potential early interventions, including symptomatic treatment of cognitive functions at early stages to further delay IADL impairment and improve patients' quality of life [53].

## Supporting information

S1 Table Serious game data. Main duration time (in minutes) per patients for each task.
(PDF)
