# Supplementary material for: Evaluation of a novel Serious Game based assessment tool for patients with Alzheimer’s disease
Source: PLoS One. 2017 May 4;12(5):e0175999. doi: 10.1371/journal.pone.0175999 (PMC5417424; doi:10.1371/journal.pone.0175999)
Supplement: S1 Table — Main duration time (in minutes) per patients for each task. (PDF) [file pone.0175999.s001.pdf]

Table 1. Serious Game data. Main duration time (in minutes) per patients for each task.

Patients	goToShop	goHome	shopping	cooking	tablePreparation	goOutside
0	3.04	2.47	0.64	9.25	1.78	0.41
0	4.18	2.79	0.38	2.27	3.01	0.22
0	3.48	2.78	0.10	3.04	1.14	0.74
0	3.80	4.08	0.56	2.54	1.56	0.47
0	3.63	5.68	0.32	3.80	1.01	0.58
0	2.99	3.81	0.24	3.84	5.42	0.54
0	4.47	2.76	0.24	2.51	2.31	0.50
0	4.97	3.42	0.39	3.95	1.13	0.67
0	3.23	2.66	0.23	2.20	2.02	0.57
0	4.90	3.52	0.30	1.98	1.36	0.47
0	3.50	2.67	0.36	4.40	1.13	0.82
0	5.57	3.40	0.25	3.14	1.48	0.28
0	3.43	2.77	0.31	5.36	2.25	0.85
0	2.77	2.27	0.18	1.95	0.72	0.21
0	5.27	12.30	0.23	2.81	4.57	0.88
1	4.64	3.06	1.46	5.37	2.55	0.57
1	5.98	6.13	1.00	5.61	2.27	0.97
1	5.79	8.20	1.43	3.49	1.54	0.73
1	8.99	5.32	0.95	5.40	2.73	0.08
1	2.58	4.66	1.36	3.45	1.84	0.49
1	5.61	6.22	1.03	5.62	1.70	0.70
0	3.11	2.65	0.14	3.06	0.75	0.35
0	2.26	2.67	0.34	2.72	1.79	0.21
1	8.75	5.92	4.10	6.76	2.45	0.84
0	3.34	3.20	0.48	3.02	1.01	0.43
1	7.27	5.18	1.99	9.49	3.37	0.82
1	3.50	5.54	1.03	2.87	1.55	0.41
1	5.61	5.41	0.53	1.05	0.94	0.43
1	3.43	4.19	3.08	4.33	1.74	0.85
0	2.20	2.12	0.46	1.32	0.68	0.16
1	2.63	3.84	0.74	2.92	2.54	0.47
1	7.56	5.89	1.06	4.46	2.67	0.46
1	8.47	8.05	1.19	5.26	2.88	0.88
1	4.16	4.21	1.87	3.27	2.15	0.53
1	3.62	4.29	2.32	7.12	2.32	0.58
1	4.87	6.47	1.31	4.24	1.96	0.58
0	2.79	2.77	0.61	2.12	1.02	0.31
1	2.90	3.99	0.59	1.54	1.25	0.77
